# Development and External Validation of an Interpretable Machine Learning‐Based Prediction Model for Depressive Symptoms in Patients With Obstructive Sleep Apnea: A Multicenter Study

**DOI:** 10.1002/brb3.71399

**Published:** 2026-04-23

**Authors:** Enguang Li, Botang Guo, Fangzhu Ai, Kuo Wen, Yangyang Tong, Ping Tang, Hongjuan Wen

**Affiliations:** ^1^ College of Management Changchun University of Chinese Medicine Changchun Jilin People's Republic of China; ^2^ Department of General Practice The Affiliated Luohu Hospital of Shenzhen University Medical School Shenzhen Guangdong People's Republic of China; ^3^ School of Nursing Jinzhou Medical University Jinzhou Liaoning People's Republic of China; ^4^ College of Traditional Chinese Medicine Changchun University of Chinese Medicine Changchun Jilin People's Republic of China; ^5^ Department of Pulmonary Oncology Affiliated Hospital of Changchun University of Chinese Medicine Changchun Jilin People's Republic of China

**Keywords:** depressive symptoms, machine learning, obstructive sleep apnea, prediction model

## Abstract

**Introduction:**

Depressive symptoms commonly co‐occur with obstructive sleep apnea (OSA), increase disease burden, and may precede major depressive disorder (MDD). In routine sleep‐clinic practice, mood symptoms can be overlooked during the work‐up for suspected OSA and at the time of polysomnography (PSG)‐confirmed diagnosis, because consultations focus primarily on sleep‐breathing and cardiometabolic complaints. Guided by the biopsychosocial model, this multicenter study aimed to develop and validate an interpretable machine‐learning (ML) model to predict the risk of depressive symptoms in patients with OSA.

**Methods:**

This study included 634 adults with OSA from two sleep centers. Participants from the first center (*n* = 400) were randomly allocated to a training cohort and an internal validation cohort in a 7:3 ratio. An external validation cohort (*n* = 234) was recruited from the second center. Depressive symptoms were defined as a Patient Health Questionnaire‑9 (PHQ‑9) score ≥ 10. Candidate predictors covered biological, psychological, and social factors. Least absolute shrinkage and selection operator (LASSO) regression was used for feature selection. Eight ML algorithms were trained and tuned by 10‑fold cross‑validation. The best‑performing model was interpreted using SHapley Additive exPlanations (SHAP), and a web‑based prediction tool was constructed.

**Results:**

In the external validation cohort, the random forest (RF) model showed the best overall performance, with an area under the receiver operating characteristic curve (AUC) of 0.815, accuracy of 0.833, and Brier score of 0.154. Decision‑curve analysis supported its clinical utility. SHAP analysis identified perceived stress level, apnea–hypopnea index (AHI), and hypertension as the most influential predictors, followed by OSA severity, sleep quality, total sleep time, mean oxygen saturation (MSaO_2_), body mass index (BMI), and sex.

**Conclusion:**

This study developed and externally validated an interpretable random forest‐based model to predict the risk of depressive symptoms in patients with OSA. The model integrates key biopsychosocial features, shows good discrimination and calibration, and offers a favorable net benefit. The accompanying web‑based tool supports practical risk assessment and may facilitate early identification, risk stratification, and personalized intervention to help prevent progression to MDD.

## Graphical abstract

This multicenter study developed and externally validated an interpretable random forest model to predict depressive symptoms in patients with obstructive sleep apnea (OSA). By integrating biopsychosocial factors, the model identified perceived stress, AHI, and hypertension as top predictors. The resulting web‐based tool enhances clinical transparency and facilitates early, personalized interventions in routine sleep clinics.

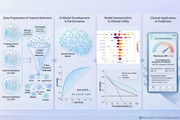

## 1 Introduction

Obstructive sleep apnea (OSA) is a common sleep‐related breathing disorder whose consequences extend beyond nocturnal respiratory disturbance. OSA is associated with cardiovascular disease, metabolic syndrome, and neurocognitive impairment, which together increase the risk of adverse clinical outcomes (Giampá et al. 2023; Oliver et al. 2024; Yeghiazarians et al. 2021). Depressive symptoms are also highly prevalent in individuals evaluated for or diagnosed with OSA and may progress to major depressive disorder (MDD); epidemiological studies suggest that about one‐third of people with OSA report clinically significant depressive symptoms, a proportion higher than in the general population (Li et al. 2023). Importantly, co‐occurrence does not imply a simple one‐way causal pathway. OSA is often long‐standing and may develop over decades before diagnosis, whereas vulnerability to depression can arise earlier in life. The two conditions may share cardiometabolic and neurobiological determinants. Serotonergic modulation, a core feature of depressive disorders and their treatment, has been linked to altered sleep‐related breathing and nocturnal oxygenation in depression, and acute serotonin reuptake inhibition can modify central chemoreflex control of ventilation. Whether these pathways causally contribute to sleep‐disordered breathing remains to be determined (Carr et al. 2025; Robillard et al. 2021).

Depressive symptoms cause psychological distress and are widely regarded as a prodromal stage of MDD. If left unrecognized and untreated, they may evolve into full‑syndrome MDD, which is difficult to treat and associated with frequent relapse and marked functional impairment (Li et al. 2024; Zarza‐Rebollo et al. 2024). By contrast, the early stage of depressive symptoms provides an important opportunity for intervention. Targeted strategies—such as psychological support, stress management, and optimization of OSA treatment—may alleviate emotional distress, prevent transition to MDD, and interrupt the vicious cycle in which depressive symptoms reduce quality of life and treatment adherence and thereby worsen OSA (Cui et al. 2024; Faye et al. 2015; Shell et al. 2024).

In contemporary sleep‐clinic practice, depressive symptoms among patients evaluated for suspected OSA are commonly assessed using validated self‐report questionnaires and, when available, structured psychiatric interviews (Björnsdóttir et al. 2016; Niraula et al. 2024). Although useful for screening, these approaches have several limitations: scores may be influenced by transient mood and by symptom overlap between depression and sleep‐disordered breathing; comprehensive psychological evaluation is difficult to implement in busy clinics; and detection depends heavily on patients’ insight and willingness to disclose symptoms. Consequently, early depressive symptoms may be missed at the time of OSA work‐up or shortly after PSG‐confirmed diagnosis. Moreover, associations between OSA severity indices and depressive symptoms are not always consistent, suggesting that physiological severity alone does not fully explain psychological risk (Vanek et al. 2020).

Machine learning (ML) offers a framework for early risk prediction of depressive symptoms. ML models can integrate multidimensional data—including biological, psychological, and social variables—and capture nonlinear relationships and higher‑order interactions, thereby improving predictive performance (Li et al. 2020). Despite growing use of ML in mental‑health research, few studies have specifically targeted depressive‑symptom prediction in people with OSA, and many existing models lack external validation.

Another barrier to clinical adoption is the perception of ML models as “black boxes” with opaque decision‐making processes that erode clinicians’ trust (Ghassemi et al. 2021). Explainable artificial‑intelligence techniques such as SHAP quantify the contribution of each feature to individual predictions while maintaining high accuracy, thereby improving interpretability and clinical credibility (Li et al. 2022).

In this context, this study aimed to develop and validate an interpretable machine‐learning model, grounded in the biopsychosocial framework, to identify patients with OSA at increased risk of depressive symptoms at the time of sleep‐clinic assessment. Using multicenter data, this study trained and externally validated several machine‐learning models, evaluated discrimination, calibration, and clinical utility, and selected the best‐performing model. SHAP was then applied to provide global and local interpretability, and an online prediction tool was developed to support triage, targeted mood screening, risk stratification, and tailored interventions in routine practice.

## 2 Materials and Methods

### 2.1 Study Design and Participants

This multicenter observational study was conducted from January to May 2025 at the Sleep Monitoring Centre of the Department of Pulmonary Oncology, Affiliated Hospital of Changchun University of Chinese Medicine. Although this sleep center is administratively located within a pulmonary oncology department, recruitment targeted adults referred for evaluation of sleep‐disordered breathing rather than for oncologic treatment. A total of 400 patients with OSA were enrolled by convenience sampling and randomly assigned, in a 7:3 ratio, to a training cohort and an internal validation cohort.

To assess generalizability, an external validation cohort of 234 consecutive patients with OSA was recruited from June to October 2025 at the Sleep Monitoring Centre of Luohu Hospital, Affiliated Hospital of Shenzhen University Medical School. Data from this cohort were used for independent external validation of the final model. The two recruitment periods occurred in different seasons; therefore, external validation across these periods helped to assess the model's robustness to potential seasonal variation in sleep and mood.

Inclusion criteria were: age ≥ 18 years; voluntary participation with written informed consent; intact consciousness and adequate communication and comprehension; OSA diagnosed by overnight polysomnography (PSG) according to clinical practice guidelines, with AHI > 5 events/h (Akashiba et al. 2022); and no prior treatment with continuous positive airway pressure (CPAP) or documented CPAP intolerance. Exclusion criteria were: active malignancy or other severe somatic disease; severe psychiatric disorders or confirmed cognitive impairment; pregnancy or lactation; nasal or sinus conditions severely affecting upper‐airway patency or a history of nasopharyngeal or oropharyngeal surgery; and concomitant organic or respiratory diseases that could markedly affect sleep architecture. Thus, although one sleep center was housed within a pulmonary oncology department, patients with diagnosed malignancy were not included in the final study cohorts. In the final analytic sample, no participant had a diagnosed malignancy (0/634, 0.0%).

Overnight PSG was performed and scored according to standard criteria. Respiratory events were scored by trained technologists following the American Academy of Sleep Medicine (AASM) recommended rules. Apnea was defined as a ≥90% drop in airflow lasting ≥10 s. Hypopnea was defined as a ≥30% reduction in airflow lasting ≥10 s accompanied by ≥3% oxygen desaturation and/or an EEG arousal. The AHI (events/h) was calculated as the total number of apneas and hypopneas divided by total sleep time. OSA severity was classified as mild (5 ≤ AHI < 15), moderate (15 ≤ AHI < 30), or severe (AHI ≥ 30). To improve comparability, both centers used the same event‐scoring definitions and underwent standardized training before data collection.

### 2.2 Sample Size

Sample size was determined using the events‑per‑variable approach for prediction modeling (Peduzzi et al. 1996). At least 10 outcome events per predictor were targeted. This study anticipated 8–10 predictors in the final model and used nine predictors for the sample‐size calculation. Assuming a depressive‐symptom prevalence of 31.9% among adults with OSA (Li et al. 2023) and allowing for 10% invalid questionnaires, the minimum required sample size was (10 × 9) / 0.319 / 0.9 ≈ 313. The final sample of 634 patients exceeded this requirement.

### 2.3 Outcome Variable

Depressive symptoms were assessed using the Patient Health Questionnaire‑9 (PHQ‑9), a self‑report instrument based on DSM‑IV criteria (Spitzer et al. 1999) and validated in Chinese populations (Kroenke et al. 2010). Each of the nine items is rated from 0 (“not at all”) to 3 (“nearly every day”), yielding a total score of 0–27. A score ≥ 10 was used to define clinically significant depressive symptoms (Li et al. 2025). In this study, Cronbach's α for the PHQ‑9 was 0.923.

### 2.4 Predictor Variables

Candidate predictors were identified through literature review, expert consultation, and clinical experience and were categorized according to the biopsychosocial model.

Biological variables included age, sex, BMI, AHI, Oxygen Desaturation Index (ODI), mean and lowest oxygen saturation (MSaO_2_, LSaO_2_), total sleep time, OSA severity, duration of OSA, hypertension, diabetes, cardiovascular disease, heart disease, and hyperlipidemia. Sociodemographic and baseline clinical data were collected via a structured questionnaire.

Psychological variables included anxiety symptoms, perceived stress, daytime sleepiness, and sleep quality. Anxiety was measured using the Generalized Anxiety Disorder 7‑Item Scale (GAD‑7) (Kertz et al. 2013; Spitzer et al. 2006). Daytime sleepiness was assessed with the Epworth Sleepiness Scale (ESS) (Johns 1991). Perceived stress was measured using the Chinese Perceived Stress Scale (CPSS) (Levenstein et al. 1993; Yang and Huang 2003). Sleep quality was assessed with the Pittsburgh Sleep Quality Index (PSQI) (Buysse et al. 1989).

Social variables included education level, monthly income, marital status, smoking status, alcohol consumption, weekly exercise, and social support measured by the Social Support Rating Scale (SSRS) (Wang et al. 2024; Xiao 1994). All instruments have been widely used in Chinese populations and showed high internal consistency in this study.

Objective physiological indicators (BMI, AHI, ODI, total sleep time, MSaO_2_, LSaO_2_, OSA severity) were obtained from overnight PSG and routine clinical assessments using standardized procedures and calibrated equipment.

### 2.5 Statistical Analysis and Feature Screening

All analyses were conducted using R. Normally distributed continuous variables are presented as mean ± standard deviation (SD), non‑normally distributed variables as median and interquartile range [M (Q_1_, Q_3_)], and categorical variables as *n* (%). Between‑group comparisons used the chi‑square test for categorical variables, the independent‑samples t‑test for normally distributed continuous variables, and the Mann–Whitney *U* test for non‑normally distributed variables. Two‐sided *p* values < 0.05 were considered statistically significant. Given the potential collinearity among PSG‐derived indicators, this study prioritized regularization and tree‐based methods that are robust to correlated predictors and applied SHAP to enhance model interpretability.

Feature selection and model construction were based on the training cohort. LASSO regression was used for variable selection, with 10‑fold cross‑validation to determine the optimal penalty parameter *λ*. Variables with non‑zero coefficients at λ.min were retained as candidate predictors.

### 2.6 Model Construction and Evaluation

Using the selected predictors, this study constructed eight ML‑based models: logistic regression (LR), support vector machine (SVM), gradient boosting machine (GBM), neural network, random forest (RF), extreme gradient boosting (XGBoost), k‑nearest neighbors (KNN), and light gradient boosting machine (LightGBM). For each algorithm, hyperparameters were tuned using grid search with 10‑fold cross‑validation in the training cohort.

Model performance in the internal and external validation cohorts was evaluated in terms of discrimination, calibration, and clinical utility. Discrimination was assessed using the AUC and concordance index (C‑index). Additional metrics included accuracy, recall (sensitivity), specificity, precision (positive predictive value), and the F1 score. Calibration was examined using calibration plots and the Brier score. Clinical utility was evaluated using decision‑curve analysis (DCA), which compares net benefit across a range of risk thresholds with “treat all” and “treat none” strategies.

To enhance interpretability, SHAP was applied to the best‑performing model. SHAP values quantify the contribution of each feature to individual predictions and to overall model output, providing global and local explanations through beeswarm, importance, waterfall, and force plots.

### 2.7 Web‑Based Prediction Tool

An interactive web‑based application was developed using the Shiny package in R, based on the final RF model. The tool allows clinicians to enter nine predictor variables and returns an estimated probability of depressive symptoms, thereby supporting risk assessment and clinical decision‑making.

### 2.8 TRIPOD‑AI Statement

Model development, validation, and reporting followed the Transparent Reporting of a Multivariable Prediction Model for Individual Prognosis or Diagnosis—Artificial Intelligence Extension (TRIPOD‑AI) guidelines (Collins et al. 2024).

## 3 Results

### 3.1 Baseline Characteristics

Using a fixed random seed (58), 400 patients from the first center were randomly divided into a training cohort (*n* = 280) and an internal validation cohort (*n* = 120). No statistically significant differences in baseline characteristics were observed between the two cohorts (all *p* > 0.05), indicating good balance (Table 1).

**TABLE 1:** Comparison of baseline data between training cohort and internal validation cohort.

Variables	Training cohort (*n* = 280)	Internal validation cohort (*n* = 120)	Statistic	*p*
Age, mean ± SD	44.60 ± 11.91	45.43 ± 11.82	*t* = 0.64	0.521
Gender, *n*(%)			*χ* ^2^ = 0.80	0.372
Male	251 (89.64)	111 (92.50)		
Female	29 (10.36)	9 (7.50)		
Education level, *n*(%)			*χ* ^2^ = 2.38	0.305
Primary school	78 (27.86)	41 (34.17)		
Junior high school	86 (30.71)	29 (24.17)		
High school and above	116 (41.43)	50 (41.67)		
Marital status, *n*(%)			*χ* ^2^ = 1.14	0.767
Married	165 (58.93)	74 (61.67)		
Unmarried	54 (19.29)	18 (15.00)		
Divorced	39 (13.93)	17 (14.17)		
Widowed	22 (7.86)	11 (9.17)		
Income level, *n*(%), yuan			*χ* ^2^ = 2.81	0.421
<3000	117 (41.79)	54 (45.00)		
3000–5000	51 (18.21)	16 (13.33)		
5000–8000	75 (26.79)	38 (31.67)		
>8000	37 (13.21)	12 (10.00)		
BMI, *n*(%)			*χ* ^2^ = 1.80	0.406
Normal	58 (20.71)	28 (23.33)		
Overweight	71 (25.36)	36 (30.00)		
Obese	151 (53.93)	56 (46.67)		
Smoking status, *n*(%)			*χ* ^2^ = 0.19	0.910
Never	74 (26.43)	33 (27.50)		
Ever	102 (36.43)	41 (34.17)		
Current	104 (37.14)	46 (38.33)		
Alcohol consumption, *n*(%)			*χ* ^2^ = 1.91	0.385
Never	77 (27.50)	27 (22.50)		
Occasional	65 (23.21)	25 (20.83)		
Everyday	138 (49.29)	68 (56.67)		
Weekly exercise status, *n*(%)			*χ* ^2^ = 1.43	0.488
Barely	141 (50.36)	53 (44.17)		
Occasionally	111 (39.64)	52 (43.33)		
Regularly	28 (10.00)	15 (12.50)		
Hypertension, *n*(%)			*χ* ^2^ = 0.00	0.965
Yes	158 (56.43)	68 (56.67)		
No	122 (43.57)	52 (43.33)		
Diabetes, *n*(%)			*χ* ^2^ = 1.69	0.194
Yes	36 (12.86)	10 (8.33)		
No	244 (87.14)	110 (91.67)		
Heart disease, *n*(%)			*χ* ^2^ = 1.21	0.271
Yes	55 (19.64)	18 (15.00)		
No	225 (80.36)	102 (85.00)		
Cerebrovascular disease, *n*(%)			*χ* ^2^ = 0.14	0.705
Yes	56 (20.00)	26 (21.67)		
No	224 (80.00)	94 (78.33)		
Hyperlipidemia, *n*(%)			*χ* ^2^ = 1.31	0.253
Yes	103 (36.79)	37 (30.83)		
No	177 (63.21)	83 (69.17)		
OSA severity, *n*(%)			*χ* ^2^ = 2.08	0.353
Mild	53 (18.93)	25 (20.83)		
Moderate	129 (46.07)	46 (38.33)		
Severe	98 (35.00)	49 (40.83)		
Anxiety symptoms, *n*(%)			*χ* ^2^ = 3.67	0.299
No	53 (18.93)	20 (16.67)		
Mild	64 (22.86)	33 (27.50)		
Moderate	83 (29.64)	42 (35.00)		
Severe	80 (28.57)	25 (20.83)		
Daytime sleepiness, *n*(%)			*χ* ^2^ = 1.76	0.625
Normal	89 (31.79)	31 (25.83)		
Mild–moderate sleepiness	47 (16.79)	24 (20.00)		
Excessive sleepiness	89 (31.79)	42 (35.00)		
Severe/dangerous sleepiness	55 (19.64)	23 (19.17)		
Perceived stress level, *n*(%)			*χ* ^2^ = 2.43	0.296
Slight	81 (28.93)	43 (35.83)		
Obvious	114 (40.71)	48 (40.00)		
Excessive	85 (30.36)	29 (24.17)		
Social support, *n*(%)			*χ* ^2^ = 2.33	0.311
Low	67 (23.93)	21 (17.50)		
Medium	69 (24.64)	29 (24.17)		
High	144 (51.43)	70 (58.33)		
Sleep quality, *n*(%)			*χ* ^2^ = 0.13	0.718
Bad	198 (70.71)	87 (72.50)		
Good	82 (29.29)	33 (27.50)		
OSA disease course, M (Q_1_, Q_3_)	15.00 (10.00, 20.00)	15.00 (10.00, 20.00)	*Z* = −1.09	0.277
AHI, M (Q_1_, Q_3_)	27.05 (21.32, 43.20)	27.70 (21.45, 47.52)	*Z* = −0.61	0.544
ODI, M (Q_1_, Q_3_)	24.50 (18.32, 40.30)	25.20 (17.95, 44.03)	*Z* = −0.53	0.597
Total sleep time, M (Q_1_, Q_3_)	408.30 (393.25, 488.50)	408.65 (394.57, 468.62)	*Z* = −0.15	0.883
MSaO_2_, M (Q_1_, Q_3_)	92.80 (90.27, 94.10)	93.00 (90.20, 94.00)	*Z* = −0.18	0.859
LSaO_2_, M (Q_1_, Q_3_)	76.00 (67.00, 83.00)	75.00 (65.75, 82.00)	*Z* = −0.59	0.557
Depressive symptoms, *n*(%)			*χ* ^2^ = 0.16	0.692
Yes	92 (32.86)	37 (30.83)		
No	188 (67.14)	83 (69.17)		

Abbreviations: AHI, apnea–hypopnea index; BMI, body mass index; LSaO_2_, lowest oxygen saturation; M, median; MSaO_2_, mean oxygen saturation; ODI, oxygen desaturation index; OSA, obstructive sleep apnea; Q_1_, first quartile; Q_3_, third quartile; SD, standard deviation; t, *t*‐test; *Z*, Mann–Whitney test; *χ* ^2^, chi‐square test.

Within the training cohort, 92 patients, representing 32.9% of the sample, had depressive symptoms, defined as a PHQ‐9 score of at least 10. Compared with the 188 patients without depressive symptoms, these patients were more likely to be divorced or widowed and had higher prevalences of hypertension, cerebrovascular disease, and hyperlipidemia; all comparisons were significant with *p* < 0.05. They also had more severe OSA, reflected by a higher proportion of severe OSA, higher median AHI and ODI values, and lower LSaO_2_; each difference was significant with *p* < 0.01. Notably, depressive symptoms were not observed among patients with mild OSA, whereas prevalence increased across severity categories, occurring in 37 of 129 patients with moderate OSA and 55 of 98 patients with severe OSA, as shown in Table 2. In addition, patients with depressive symptoms reported more severe anxiety, greater daytime sleepiness, higher perceived stress, and poorer sleep quality; all differences were significant with *p* < 0.01 and are summarized in Table 2.

**TABLE 2:** Distribution of baseline data in the training cohort.

Variables	Total data (*n* = 280)	Depressive symptoms (*n* = 92)	Non‐depressive symptoms (*n* = 188)	Statistic	*P*
Age, mean ± SD	44.60 ± 11.91	45.87 ± 12.12	43.98 ± 11.78	*t* = −1.25	0.213
Gender, *n*(%)				*χ* ^2^ = 0.41	0.523
Male	251 (89.64)	84 (91.30)	167 (88.83)		
Female	29 (10.36)	8 (8.70)	21 (11.17)		
Education level, *n*(%)				*χ* ^2^ = 3.94	0.140
Primary school	78 (27.86)	19 (20.65)	59 (31.38)		
Junior high school	86 (30.71)	29 (31.52)	57 (30.32)		
High school and above	116 (41.43)	44 (47.83)	72 (38.30)		
Marital status, *n*(%)				*χ* ^2^ = 8.00	**0.046**
Married	165 (58.93)	52 (56.52)	113 (60.11)		
Unmarried	54 (19.29)	12 (13.04)	42 (22.34)		
Divorced	39 (13.93)	19 (20.65)	20 (10.64)		
Widowed	22 (7.86)	9 (9.78)	13 (6.91)		
Income level, *n*(%), yuan				*χ* ^2^ = 2.96	0.398
<3000	117 (41.79)	44 (47.83)	73 (38.83)		
3000–5000	51 (18.21)	13 (14.13)	38 (20.21)		
5000–8000	75 (26.79)	22 (23.91)	53 (28.19)		
>8000	37 (13.21)	13 (14.13)	24 (12.77)		
BMI, n(%)				*χ* ^2^ = 2.27	0.321
Normal	58 (20.71)	15 (16.30)	43 (22.87)		
Overweight	71 (25.36)	22 (23.91)	49 (26.06)		
Obese	151 (53.93)	55 (59.78)	96 (51.06)		
Smoking status, *n*(%)				*χ* ^2^ = 0.44	0.802
Never	74 (26.43)	23 (25.00)	51 (27.13)		
Ever	102 (36.43)	36 (39.13)	66 (35.11)		
Current	104 (37.14)	33 (35.87)	71 (37.77)		
Alcohol consumption, *n*(%)				*χ* ^2^ = 0.43	0.806
Never	77 (27.50)	23 (25.00)	54 (28.72)		
Occasional	65 (23.21)	22 (23.91)	43 (22.87)		
Everyday	138 (49.29)	47 (51.09)	91 (48.40)		
Weekly exercise status, *n*(%)				*χ* ^2^ = 5.90	0.052
Barely	141 (50.36)	53 (57.61)	88 (46.81)		
Occasionally	111 (39.64)	35 (38.04)	76 (40.43)		
Regularly	28 (10.00)	4 (4.35)	24 (12.77)		
Hypertension, *n*(%)				*χ* ^2^ = 21.54	**<0.001**
Yes	158 (56.43)	70 (76.09)	88 (46.81)		
No	122 (43.57)	22 (23.91)	100 (53.19)		
Diabetes, *n*(%)				*χ* ^2^ = 0.20	0.656
Yes	36 (12.86)	13 (14.13)	23 (12.23)		
No	244 (87.14)	79 (85.87)	165 (87.77)		
Heart disease, *n*(%)				*χ* ^2^ = 1.58	0.208
Yes	55 (19.64)	22 (23.91)	33 (17.55)		
No	225 (80.36)	70 (76.09)	155 (82.45)		
Cerebrovascular disease, *n*(%)				*χ* ^2^ = 7.48	**0.006**
Yes	56 (20.00)	27 (29.35)	29 (15.43)		
No	224 (80.00)	65 (70.65)	159 (84.57)		
Hyperlipidemia, *n*(%)				*χ* ^2^ = 4.63	**0.031**
Yes	103 (36.79)	42 (45.65)	61 (32.45)		
No	177 (63.21)	50 (54.35)	127 (67.55)		
OSA severity, *n*(%)				*χ* ^2^ = 51.00	**<0.001**
Mild	53 (18.93)	0 (0.00)	53 (28.19)		
Moderate	129 (46.07)	37 (40.22)	92 (48.94)		
Severe	98 (35.00)	55 (59.78)	43 (22.87)		
Anxiety symptoms, *n*(%)				*χ* ^2^ = 12.23	**0.007**
No	53 (18.93)	7 (7.61)	46 (24.47)		
Mild	64 (22.86)	22 (23.91)	42 (22.34)		
Moderate	83 (29.64)	34 (36.96)	49 (26.06)		
Severe	80 (28.57)	29 (31.52)	51 (27.13)		
Daytime sleepiness, *n*(%)				*χ* ^2^ = 59.54	**<0.001**
Normal	89 (31.79)	11 (11.96)	78 (41.49)		
Mild–moderate sleepiness	47 (16.79)	23 (25.00)	24 (12.77)		
Excessive sleepiness	89 (31.79)	20 (21.74)	69 (36.70)		
Severe/dangerous sleepiness	55 (19.64)	38 (41.30)	17 (9.04)		
Perceived stress level, *n*(%)				*χ* ^2^ = 75.90	**<0.001**
Slight	81 (28.93)	0 (0.00)	81 (43.09)		
Obvious	114 (40.71)	38 (41.30)	76 (40.43)		
Excessive	85 (30.36)	54 (58.70)	31 (16.49)		
Social support, *n*(%)				*χ* ^2^ = 3.13	0.209
Low	67 (23.93)	22 (23.91)	45 (23.94)		
Medium	69 (24.64)	17 (18.48)	52 (27.66)		
High	144 (51.43)	53 (57.61)	91 (48.40)		
Sleep quality, *n*(%)				*χ* ^2^ = 31.09	**<0.001**
Bad	198 (70.71)	85 (92.39)	113 (60.11)		
Good	82 (29.29)	7 (7.61)	75 (39.89)		
OSA disease course, M (Q_1_, Q_3_)	15.00 (10.00, 20.00)	12.00 (10.00, 25.00)	15.00 (10.00, 20.00)	*Z* = −0.08	0.938
AHI, M (Q_1_, Q_3_)	27.05 (21.32, 43.20)	43.05 (26.62, 63.50)	24.70 (14.28, 29.40)	*Z* = −6.82	**<0.001**
ODI, M (Q_1_, Q_3_)	24.50 (18.32, 40.30)	40.40 (23.70, 57.82)	22.10 (12.07, 26.47)	*Z* = −6.89	**<0.001**
Total sleep time, M (Q_1_, Q_3_)	408.30 (393.25, 488.50)	405.90 (396.90, 419.15)	411.00 (392.50, 501.88)	*Z* = −1.71	0.087
MSaO_2_, M (Q_1_, Q_3_)	92.80 (90.27, 94.10)	92.25 (88.70, 93.90)	92.95 (90.70, 94.20)	*Z* = −1.76	0.078
LSaO_2_, M (Q_1_, Q_3_)	76.00 (67.00, 83.00)	74.50 (63.00, 80.25)	77.00 (69.00, 84.00)	*Z* = −2.68	**0.007**

Abbreviations: AHI, apnea–hypopnea index; BMI, body mass index; LSaO_2_, lowest oxygen saturation; M, median; MSaO_2_, mean oxygen saturation; ODI, oxygen desaturation index; OSA, obstructive sleep apnea; Q_1_, first quartile; Q_3_, third quartile; SD, standard deviation; t, *t*‐test; *Z*, Mann–Whitney test; *χ* ^2^, chi‐square test.

### 3.2 Predictive Variable Screening

**FIGURE 1:**
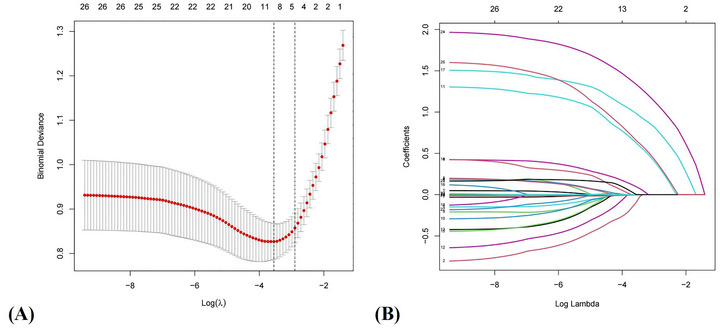
Feature variable screening process. (A) Feature selection based on LASSO algorithm. The partial likelihood deviance (binomial deviance) curve was plotted vs. log (λ). The dotted vertical lines represent the optimal predictors using the minimum criteria (λ.min) and the 1 se of the minimum criteria (λ.1se). (B) A total of nine clinical features were selected based on the coefficients (λ.min) under the LASSO algorithm.

LASSO regression with 10‑fold cross‑validation identified λ.min = 0.0288 and λ.1se = 0.0552. To maximize predictive performance, λ.min was selected, yielding nine predictors with non‑zero coefficients: sex, BMI, hypertension, AHI, OSA severity, total sleep time, MSaO_2_, perceived stress level, and sleep quality (Figure 1).

### 3.3 Model Construction and Performance Evaluation

**FIGURE 2:**
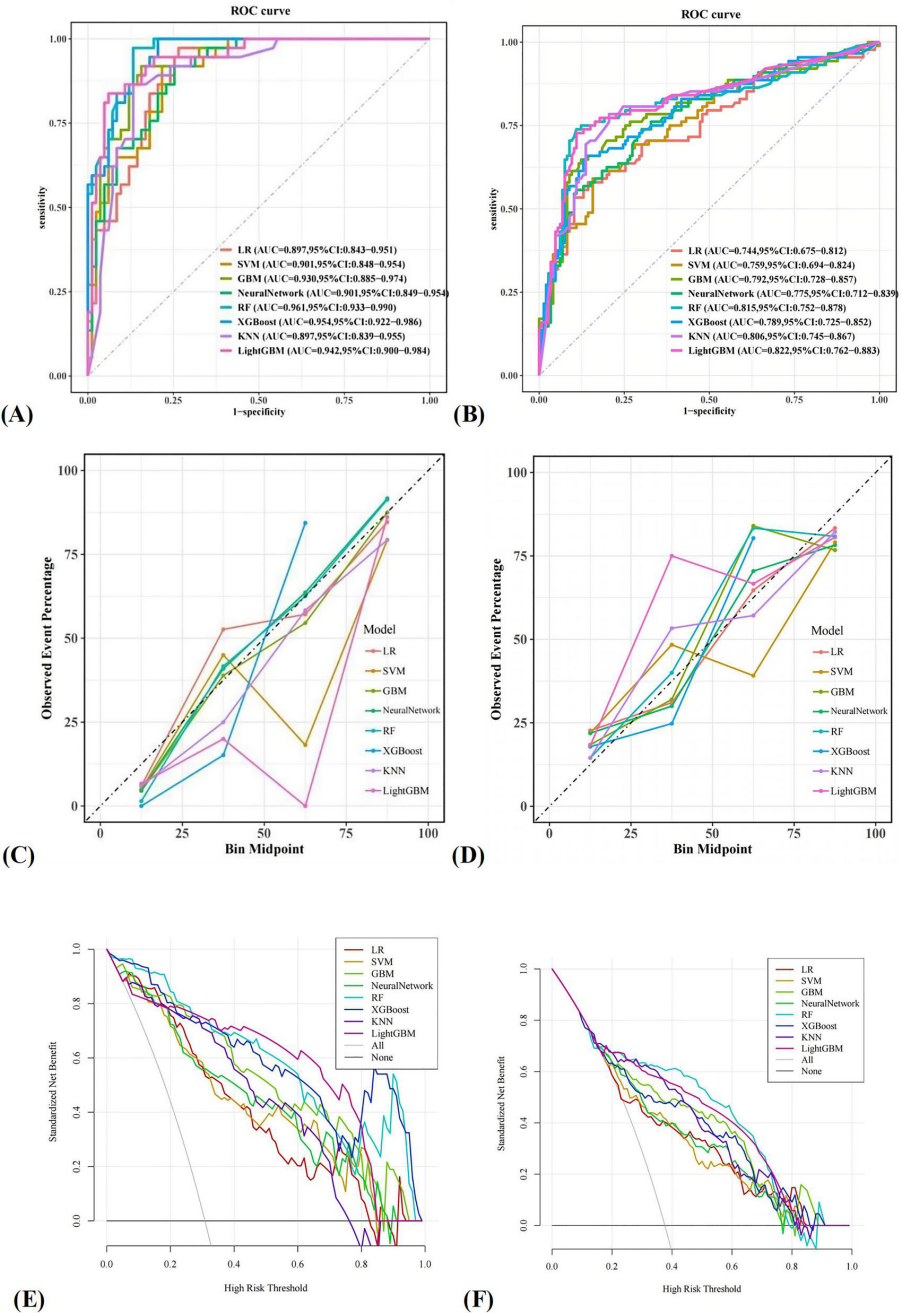
Performance comparison of eight machine learning models. (A) Comparison of ROC curves in the internal validation cohort. (B) Comparison of ROC curves on the external validation cohort. (C) Comparison of calibration curves in the internal validation cohort. (D) Comparison of calibration curves on the external validation cohort. (E) Comparison of DCA in the internal validation cohort. (F) Comparison of DCA on the external validation cohort. GBM, gradient boosting machine; KNN, K‐nearest neighbor; LightGBM, light gradient boosting machine; LR, logistic regression; RF, random forest; SVM, support vector machine; XGBoost, eXtreme gradient boosting.

Using these predictors, eight ML models—LR, SVM, GBM, neural network, RF, XGBoost, KNN, and LightGBM—were trained and evaluated (Figure 2; Table 3). Optimal hyperparameter settings for each model are provided in Supporting Information 1.

**TABLE 3:** Confusion matrix results of eight machine learning models.

Data set	Model	Accuracy	Recall	Specificity	Precision	F1 score	Brier score	C index
**Train**	LR	0.846	0.891	0.824	0.713	0.792	0.112	0.911
	SVM	0.821	0.935	0.766	0.662	0.775	0.107	0.920
	GBM	0.918	0.902	0.926	0.856	0.878	0.065	0.969
	NeuralNetwork	0.846	0.891	0.824	0.713	0.792	0.109	0.920
	RF	0.982	0.978	0.984	0.968	0.973	0.030	0.997
	XGBoost	0.911	0.848	0.941	0.876	0.862	0.136	0.956
	KNN	0.936	0.989	0.910	0.843	0.910	0.051	0.987
	LightGBM	0.986	1.000	0.979	0.958	0.979	0.006	1.000
**Internal**	LR	0.808	0.973	0.735	0.621	0.758	0.126	0.897
	SVM	0.825	0.919	0.783	0.654	0.764	0.131	0.901
	GBM	0.867	0.919	0.843	0.723	0.810	0.098	0.930
	NeuralNetwork	0.800	0.919	0.747	0.618	0.739	0.121	0.901
	RF	0.900	0.973	0.867	0.766	0.857	0.080	0.961
	XGBoost	0.858	1.000	0.795	0.685	0.813	0.136	0.954
	KNN	0.867	0.865	0.867	0.744	0.800	0.121	0.897
	LightGBM	0.908	0.838	0.940	0.861	0.849	0.097	0.942
**External**	LR	0.752	0.580	0.856	0.708	0.637	0.202	0.744
	SVM	0.748	0.591	0.842	0.693	0.638	0.196	0.759
	GBM	0.791	0.648	0.877	0.760	0.699	0.173	0.792
	NeuralNetwork	0.765	0.557	0.890	0.754	0.641	0.190	0.775
	RF	0.833	0.739	0.890	0.802	0.769	0.154	0.815
	XGBoost	0.786	0.659	0.863	0.744	0.699	0.181	0.789
	KNN	0.791	0.784	0.795	0.697	0.738	0.172	0.806
	LightGBM	0.829	0.727	0.890	0.800	0.762	0.176	0.822

Abbreviations: External, external validation cohort; GBM, gradient boosting machine; Internal, internal validation cohort; KNN, K‐nearest neighbor; LGBM, light gradient boosting machine; LR, logistic regression; RF, random forest; SVM, support vector machine; Train, training cohort; XGBoost, eXtreme gradient boosting.

In the external validation cohort, the RF model achieved the best overall performance, with an AUC and C‑index of 0.815, accuracy of 0.833, specificity of 0.890, precision of 0.802, recall of 0.739, F1 score of 0.769, and Brier score of 0.154. GBM and XGBoost showed moderate performance (AUCs 0.792 and 0.789, respectively). KNN yielded relatively high recall (0.784) but lower precision (0.697). LR and SVM performed less well (AUCs < 0.76).

LightGBM demonstrated high discrimination in the internal validation cohort (AUC = 0.942) but a larger decline in the external cohort (AUC = 0.822), suggesting overfitting. RF showed excellent discrimination internally (AUC = 0.961) and a smaller performance drop externally (AUC = 0.815), indicating better generalizability.

Calibration plots indicated that RF predictions were closest to the ideal 45° line, and RF had the lowest Brier score in the external cohort. DCA showed that RF provided the greatest net benefit across clinically relevant threshold probabilities (0.20–0.60), whereas traditional models yielded decision curves close to “treat all” or “treat none.” Overall, RF offered the most favorable combination of discrimination, calibration, and clinical utility and was selected as the final model.

### 3.4 Evaluation of RF Model Fit and Generalization

In the training cohort, the mean AUC from 10‑fold cross‑validation for the RF model was 0.91, with fold‑specific AUCs ranging from 0.837 to 1.000, indicating consistent learning without obvious underfitting. In the internal validation cohort, the AUC was 0.961. In the external validation cohort, the AUC decreased to 0.815, still indicating good discrimination but suggesting a degree of overfitting. ROC curves for these analyses are provided in Supporting Information 2.

To assess robustness to potential predictor redundancy, we conducted sensitivity analyses in the external validation cohort by alternately excluding one variable from each correlated pair (AHI vs. OSA severity; sleep quality vs. total sleep time) and refitting the RF model. Discrimination remained stable (AUCs 0.808, 0.811, 0.826, and 0.824 for the four exclusion scenarios), and calibration and net benefit were comparable across clinically relevant thresholds (Supporting Information 3).

### 3.5 Model Interpretability

**FIGURE 3:**
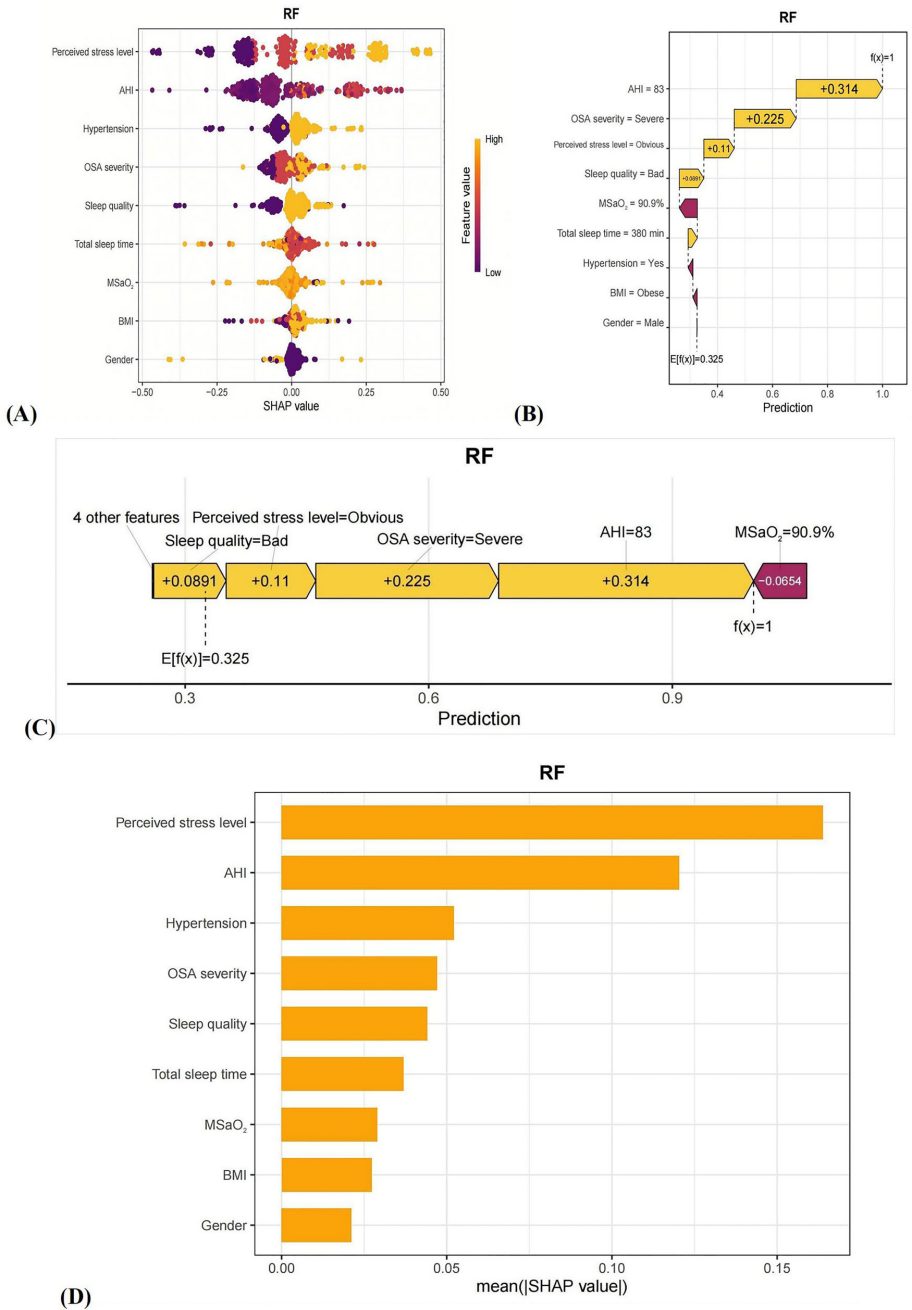
(A) SHAP beeswarm plot of the SHAP values of the model constructed by the random forest algorithm. Vertical coordinates show the importance of the features, sorted in descending order of variable importance, while the variables above are more important to the model. For horizontal positions, the "Shap value" shows whether the effect of this value is related to higher or lower predictions. The color of each SHAP value point indicates whether the observed value is high (yellow) or low (purple). (B) The waterfall plot of SHAP values for the model constructed by the random forest algorithm. (C) SHAP value force plot of the model constructed using the random forest algorithm. (D) The SHAP variable importance ranking plot of the model constructed using the random forest algorithm.

SHAP analysis provided insight into the RF model's decision‑making process (Figure 3). Perceived stress level, AHI, and hypertension were the most important predictors of depressive symptoms, followed by OSA severity, sleep quality, total sleep time, MSaO_2_, BMI, and sex. The SHAP beeswarm plot showed that high perceived stress, elevated AHI, severe OSA, poor sleep quality, and shorter total sleep time were associated with increased predicted risk. Detailed SHAP dependence plots for the three continuous predictors and six categorical predictors are provided in Supporting Information 5.

Individual‐level waterfall and force plots illustrated how each feature shifted the predicted probability from the baseline risk to the final value. In a representative high‐risk case shown in Figure 3B,C, the AHI was 83 events per hour, indicating very severe OSA. Although this value is at the extreme end, AHI is a continuous PSG‐derived metric, and such values can occur in very severe OSA; this case is shown to demonstrate model explanations at high risk. In the training cohort, AHI ranged from 6.8 to 129.5 events/h (median 27.05, IQR 21.32–43.20), and the 95th percentile was 83.13 events/h, placing this example near the upper tail but within the observed clinical spectrum (Supporting Information 4). Importantly, the model is intended for risk stratification across the typical clinical spectrum, and most participants in our cohort had moderate‐to‐severe (treatable) OSA. Along with severe OSA, high perceived stress, poor sleep quality, and short sleep duration, this profile increased the predicted probability of depressive symptoms to nearly 1. Smaller additional contributions were observed for hypertension, lower MSaO_2_, obesity, and female sex.

To provide clinically actionable context within commonly treated AHI ranges, we examined depressive‐symptom prevalence across AHI strata in the training cohort (Supporting Information 4). Prevalence increased stepwise from 0.0% for AHI 5–<15 to 28.5% for AHI 15–<30, 46.8% for AHI 30–<60, and 74.3% for AHI ≥60 (P for trend < 0.001). Within the typical treatable range, depressive‐symptom prevalence was 23.5% (42/179) for AHI 10–40 and 28.8% (42/146) for AHI 15–40.

### 3.6 Web‐Based Interactive Predictive Platform

**FIGURE 4:**
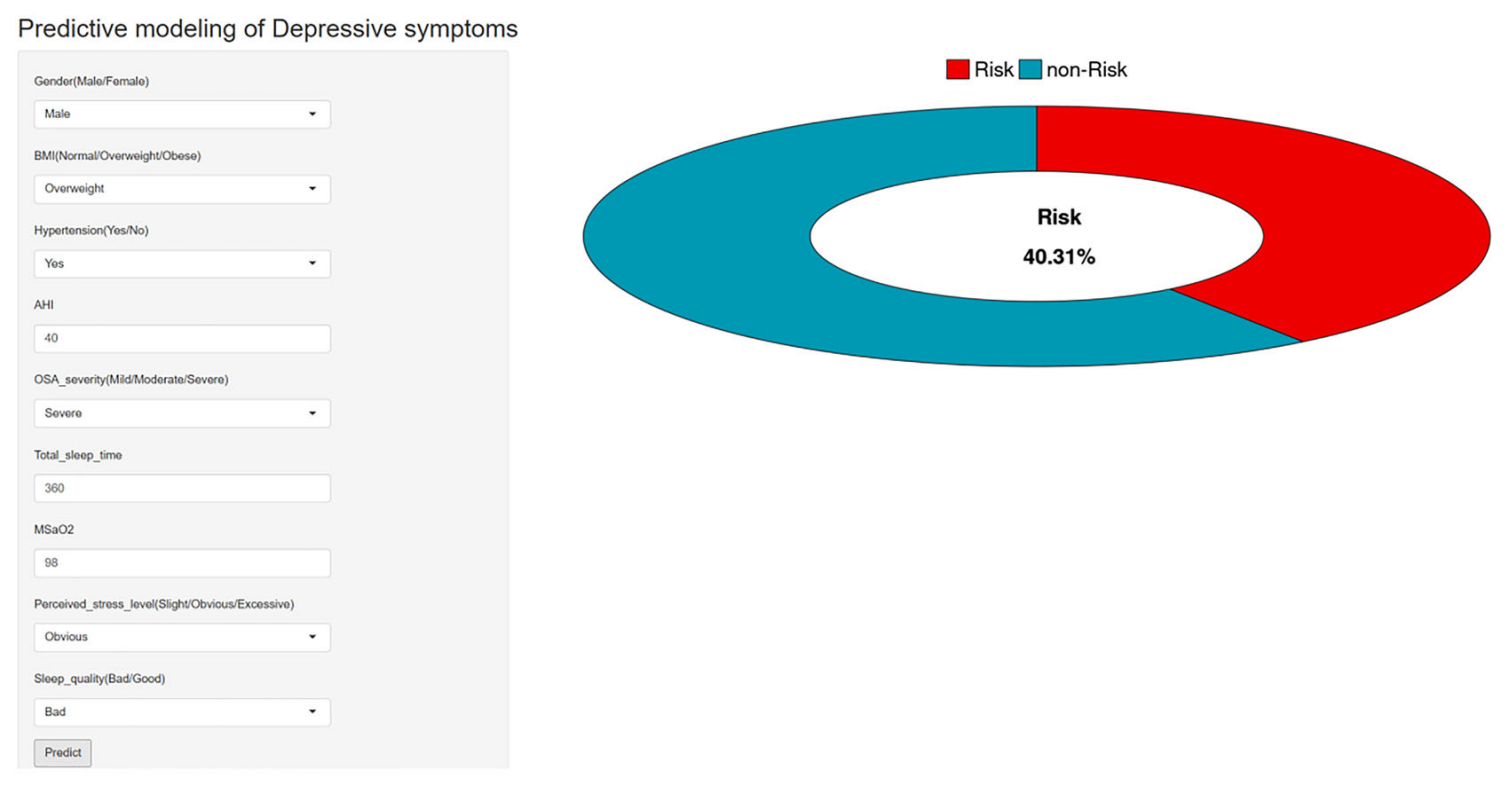
Online interactive web computing tool.

To facilitate clinical use, an online interactive prediction platform (https://depressive‐symptoms.shinyapps.io/make_web/) was developed based on the RF model (Figure 4). Clinicians can enter the nine key predictors and obtain an immediate estimate of the probability of depressive symptoms in patients with OSA. For example, for a typical patient with severe OSA—male, overweight, hypertensive, total sleep time of 360 min, MSaO_2_ of 98%, significant perceived stress, poor sleep quality, and AHI of 40—the model predicts a 40.31% probability of depressive symptoms. This estimate can be integrated with clinical judgment to guide early, individualized management.

## 4 Discussion

Using the biopsychosocial framework, this multicenter study developed and externally validated an interpretable RF‑based model to predict depressive symptoms in patients with OSA. The model showed good discrimination, calibration, and net benefit in an independent external cohort. SHAP analysis identified perceived stress, AHI, and hypertension as the strongest contributors, providing clinically intuitive explanations for individual risk estimates. A web‑based tool was created to support early identification and personalized intervention.

This study extends previous work on the OSA–depression interface by combining multicenter model development, independent external validation, and model interpretability within a single framework. By leveraging multicenter clinical data and SHAP‐based explanations, the study improves both robustness and transparency. Among the eight candidate algorithms, the random forest model showed the best overall external performance, which is consistent with the capacity of tree‐based ensemble methods to capture nonlinear relationships and complex interactions while remaining relatively robust to correlated clinical variables. Although LightGBM performed well in internal validation, its external performance was less stable, underscoring the importance of independent validation when developing clinically applicable prediction models.

Importantly, the nine retained predictors appear to operate as an interacting biopsychosocial network rather than as isolated correlates. Perceived stress may act as a proximal amplifier: in patients with high OSA burden, repeated nocturnal arousals, daytime fatigue, and illness‐related worry can increase perceived stress, while chronic stress itself is closely linked to inflammatory signaling and depression biology (Medina‐Rodriguez et al. 2023). In parallel, higher AHI and more severe OSA capture the frequency and burden of respiratory events, whereas lower MSaO_2_ and shorter total sleep time reflect their physiological consequences—intermittent hypoxemia and sleep loss. Recent evidence supports a dose‐response relationship between greater OSA severity and higher depression risk, and experimental syntheses indicate that intermittent hypoxia promotes oxidative stress, neuroinflammation, and apoptosis, providing a plausible mechanistic bridge from nocturnal breathing disturbance to depressive symptoms (El Amine et al. 2024; Zhao et al. 2024).

These physiological effects are likely to interact with cardiometabolic vulnerability. Hypertension may not simply coexist with OSA; rather, OSA‐related respiratory effort, sympathetic activation, vascular dysfunction, and inflammation are closely connected to blood‐pressure dysregulation, and those same pathways overlap with biological models of depression (Giampá et al. 2023; Martinot et al. 2023). BMI may further intensify this pathway, because obesity is strongly intertwined with OSA pathogenesis and has also been identified as a causal risk factor for depression in Mendelian‐randomization analyses (Jokela and Laakasuo 2023). Accordingly, patients with the combined profile of severe sleep‐disordered breathing, cardiometabolic comorbidity, and excess adiposity may experience a greater biological burden than would be expected from any single predictor alone.

The subjective variables also add clinically important information beyond PSG indices alone. Sleep quality may capture the lived burden of fragmented, non‐restorative sleep, whereas total sleep time reflects sleep opportunity actually achieved. Meta‐analytic evidence shows that depression is characterized by shorter total sleep time and more disrupted sleep architecture, supporting the view that subjective and objective sleep markers provide complementary affective information rather than simple redundancy (Ricciardiello et al. 2024). In the same way, perceived stress may interact with poor sleep quality to lower emotion‐regulation capacity and intensify the affective consequences of hypoxemia and fatigue; recent synthesis work linking emotion‐regulation characteristics with inflammatory activity provides a biologically plausible bridge for this interaction (Moriarity et al. 2023). This may explain why the model retained both physiological and psychological variables: together they better represent the full pathway from nocturnal respiratory dysfunction to daytime depressive symptomatology than any single PSG metric alone.

Sex may further modify how the above relationships are expressed. Large clinical cohorts have shown that men with OSA often present with higher AHI and greater oxygen desaturation, whereas women may show heavier symptom burden or poorer quality‐of‐life impairment at milder stages (Votteler et al. 2023; Wimms et al. 2024). In our cohort, the model assigned additional risk to male sex, which may reflect later presentation with greater physiological burden rather than a universal biological effect of sex itself. Taken together, these findings support the clinical interpretability of the selected predictors: the model appears to capture an interacting network of OSA burden, hypoxemia, sleep disruption, cardiometabolic vulnerability, stress, and sex‐specific phenotype.

By combining random forest modeling with SHAP and a Shiny‐based interface, this study translated complex modeling into an accessible clinical tool. The tool provides individualized risk estimates based on routinely available information, which may facilitate earlier discussion of mood symptoms, closer monitoring of high‐risk patients, and timely referral or intervention.

With respect to intended use and actionability, the tool is not designed to replace clinical assessment of mood or to diagnose depression; rather, it is intended as a decision‐support triage aid at two practical points: during the initial sleep‐clinic evaluation and after PSG‐confirmed OSA when treatment planning begins. When a patient is classified as high risk, we propose a pragmatic outpatient pathway. First, clinicians should complete depression screening with the PHQ‑9 and brief clinical questioning, because screening is most useful when diagnostic follow‐up and referral pathways are available (Barry et al. 2023). Second, immediate safety assessment is required for suicidal ideation, psychotic symptoms, or marked functional deterioration; patients with any of these red flags should receive urgent psychiatric evaluation. Third, patients without emergency features but with persistent moderate‐to‐severe depressive symptoms should be referred to mental‐health services and scheduled for early follow‐up. Fourth, OSA‐directed management should be initiated or intensified in parallel—including education, CPAP or other indicated therapy, weight management, blood pressure optimization, and sleep‐hygiene counselling—because treatment of OSA can improve depressive symptoms in at least a proportion of patients (Fu et al. 2023). Finally, for patients with high predicted risk but subthreshold screening results, repeat mood screening after PSG review or early treatment follow‐up may help identify those whose symptoms become more evident over time. For implementation, clinics may predefine a probability cut‐off that balances additional screening workload against the risk of missed cases; when the priority is case‐finding, a lower cut‐off favoring sensitivity can be selected, informed by the clinically relevant thresholds assessed in the decision‐curve analysis. From an actionability perspective, several influential predictors are potentially modifiable, including perceived stress, sleep quality, sleep duration, hypertension control, and OSA severity through effective OSA therapy, enabling the risk estimate to inform personalized follow‐up and intervention planning rather than merely assigning a risk label.

Relationship between OSA and depressive symptoms: The model targets risk at the time of adult presentation and should not be interpreted as implying that OSA onset occurred only shortly before assessment. OSA commonly remains unrecognized for years, and both OSA and depression may reflect shared long‐term trajectories shaped by early‐life neurobiological and cardiometabolic factors. In addition, depression and its neurophysiological substrates may influence respiratory control and sleep architecture. Consistent with the observational design, the findings support risk stratification and hypothesis generation rather than causal inference.

Regarding correlated predictors, AHI and the categorical OSA severity variable are closely related by definition, and subjective sleep quality can correlate with objectively measured sleep duration. In tree‐based models such as random forests, correlated features may share predictive information without substantially compromising discrimination, and SHAP can be used to visualize their contributions. This study retained both AHI and OSA severity because they provide complementary clinical interpretability: AHI captures within‐category variability, whereas severity aligns with guideline‐based treatment thresholds. Sensitivity analyses in the external validation cohort, in which we alternately excluded AHI or OSA severity and excluded sleep quality or total sleep time, yielded similar discrimination, calibration, and net benefit (Supporting Information 3), supporting robustness to potential redundancy.

Several limitations warrant consideration and inform priorities for future work. First, although the model underwent external validation, the external cohort was still modest in size (*n* = 234). Methodological studies indicate that external validation with limited sample sizes can yield imprecise estimates of discrimination and, in particular, calibration, and that performance observed in a single validation dataset may vary substantially according to case‐mix and between‐setting heterogeneity (de Winkel et al. 2024; Riley et al. 2021). Second, both cohorts were recruited from domestic sleep monitoring centers in China, and the derivation cohort came from a tertiary hospital‐based setting. The case‐mix in such centers may differ from that in other regions, cultural contexts, or primary healthcare environments, where referral thresholds, symptom presentation, comorbidity burden, and access to PSG can differ. Accordingly, even though the present results are encouraging, the model may require recalibration before use in community or primary‐care settings and in non‐Chinese populations. Third, because patients with active malignancy and other severe somatic diseases were excluded, the model is intended for non‐oncology adult OSA populations and should not be extrapolated to oncology populations without dedicated validation. Additional limitations should also be acknowledged. Participants were recruited by convenience sampling and the majority were men, which may limit generalizability to women and rural populations. The two cohorts were enrolled during different calendar periods, so seasonal influences on sleep and mood cannot be fully excluded. Depressive symptoms were assessed using the PHQ‐9 rather than structured diagnostic interviews, and additional objective markers were not incorporated. Finally, the observational design precludes causal inference and the clinical impact of the web‐based tool has not yet been tested. Future studies should therefore recruit larger and more diverse multicenter cohorts, including multinational, community‐based, and primary‐care populations, incorporate diagnostic psychiatric assessments where feasible, and evaluate recalibration, implementation, and clinical impact prospectively.

## 5 Conclusion

This multicenter study developed and externally validated an interpretable RF‐based prediction model for depressive symptoms in patients with OSA, incorporating key biological, psychological, and social variables. The model demonstrated good discrimination, calibration, and clinical net benefit in an external cohort, and SHAP‐based analyses clarified the contribution of individual predictors. The accompanying web‐based platform provides real‐time, individualized risk estimates to complement routine clinical evaluation and may support earlier recognition, risk stratification, and targeted intervention in sleep‐clinic practice. With further validation and prospective implementation testing, this approach has the potential to contribute to the proactive prevention of depression in patients with OSA.

## Author Contributions

**Enguang Li**: conceptualization, methodology, formal analysis, data curation, writing – original draft, writing – review and editing. **Botang Guo and Fangzhu Ai**: conceptualization, methodology, formal analysis, data curation, writing – original draft, writing – review and editing. **Kuo Wen**: investigation, data curation, validation. **Yangyang Tong**: software, investigation, formal analysis. **Ping Tang**: formal analysis, data curation, conceptualization, methodology, writing – original draft, writing – review and editing. **Hongjuan Wen**: conceptualization, formal analysis, supervision, writing – original draft, writing – review and editing.

## Funding

This work was supported by the Science and Technology Project of the Jilin Provincial Administration of Traditional Chinese Medicine (No. 2024260), the Changchun University of Traditional Chinese Medicine Theme Case Project (No. 2024YJ03), the Shenzhen Key Medical Discipline Construction Fund (No. SZXK062), the Shenzhen Philosophy and Social Science Planning Project (No. SZ2024C018) and 2025 Thematic Case Project of the Development Center for Degree and Graduate Education, Ministry of Education.

## Ethics Statement

Ethics approval for this study was obtained from the Ethics Committee of the Affiliated Hospital of Changchun University of Chinese Medicine (Approval No.: CCZYFYLL‐SQ‐2025‐277). All procedures were conducted in accordance with the ethical standards of the 1964 Declaration of Helsinki and its later amendments.

## Consent

Written informed consent was obtained from all participants after they had been informed about the study objectives, procedures, potential risks and benefits, and their right to withdraw at any time without penalty.

## Conflicts of Interest

The authors declare no conflicts of interest.

## Supporting information




**Supplementary Materials**: brb371399‐sup‐0001‐SuppMat.docx

**Supplementary Materials**: brb371399‐sup‐0002‐SuppMat.docx

**Supplementary Materials**: brb371399‐sup‐0003‐SuppMat.docx

**Supplementary Materials**: brb371399‐sup‐0004‐SuppMat.png

**Supplementary Materials**: brb371399‐sup‐0005‐SuppMat.pdf

**Supplementary Materials**: brb371399‐sup‐0006‐SuppMat.docx

## Data Availability

The data that support the findings of this study are available on request from the corresponding author. The data are not publicly available due to privacy or ethical restrictions.
