# Supplementary material for: Development and External Validation of an Interpretable Machine Learning‐Based Prediction Model for Depressive Symptoms in Patients With Obstructive Sleep Apnea: A Multicenter Study
Source: Brain Behav. 2026 Apr 23;16(4):e71399. doi: 10.1002/brb3.71399 (PMC13103541; doi:10.1002/brb3.71399)
Supplement: Supplementary file 1 — Supplementary Materials: brb371399‐sup‐0001‐SuppMat.docx [file BRB3-16-e71399-s006.docx]

**Best parameters for each machine learning algorithm model**

| **Model** | **Parameters** |
| --- | --- |
| GBM | n.trees=100, interaction.depth=2, shrinkage=0.1, n.minobsinnode=5 |
| KNN | kmax=13, distance=1 |
| LightGBM | min_data=1, learning_rate =1, num_threads=2, verbosity=1, num_iterations=5, early_stopping_round=3 |
| NeuralNetwork | size=4, decay=0.6 |
| RF | mtry=2 |
| SVM | Sigma=0.1, C=0.1 |
| XGBoost | Nrounds=10, max_depth=5, eta=0.1, gamma=0.5, colsample_bytree=0.5, min_child_weight=1, subsample=0.6 |
