# Supplementary material for: Development and External Validation of an Interpretable Machine Learning‐Based Prediction Model for Depressive Symptoms in Patients With Obstructive Sleep Apnea: A Multicenter Study
Source: Brain Behav. 2026 Apr 23;16(4):e71399. doi: 10.1002/brb3.71399 (PMC13103541; doi:10.1002/brb3.71399)
Supplement: Supplementary file 2 — Supplementary Materials: brb371399‐sup‐0002‐SuppMat.docx [file BRB3-16-e71399-s005.docx]

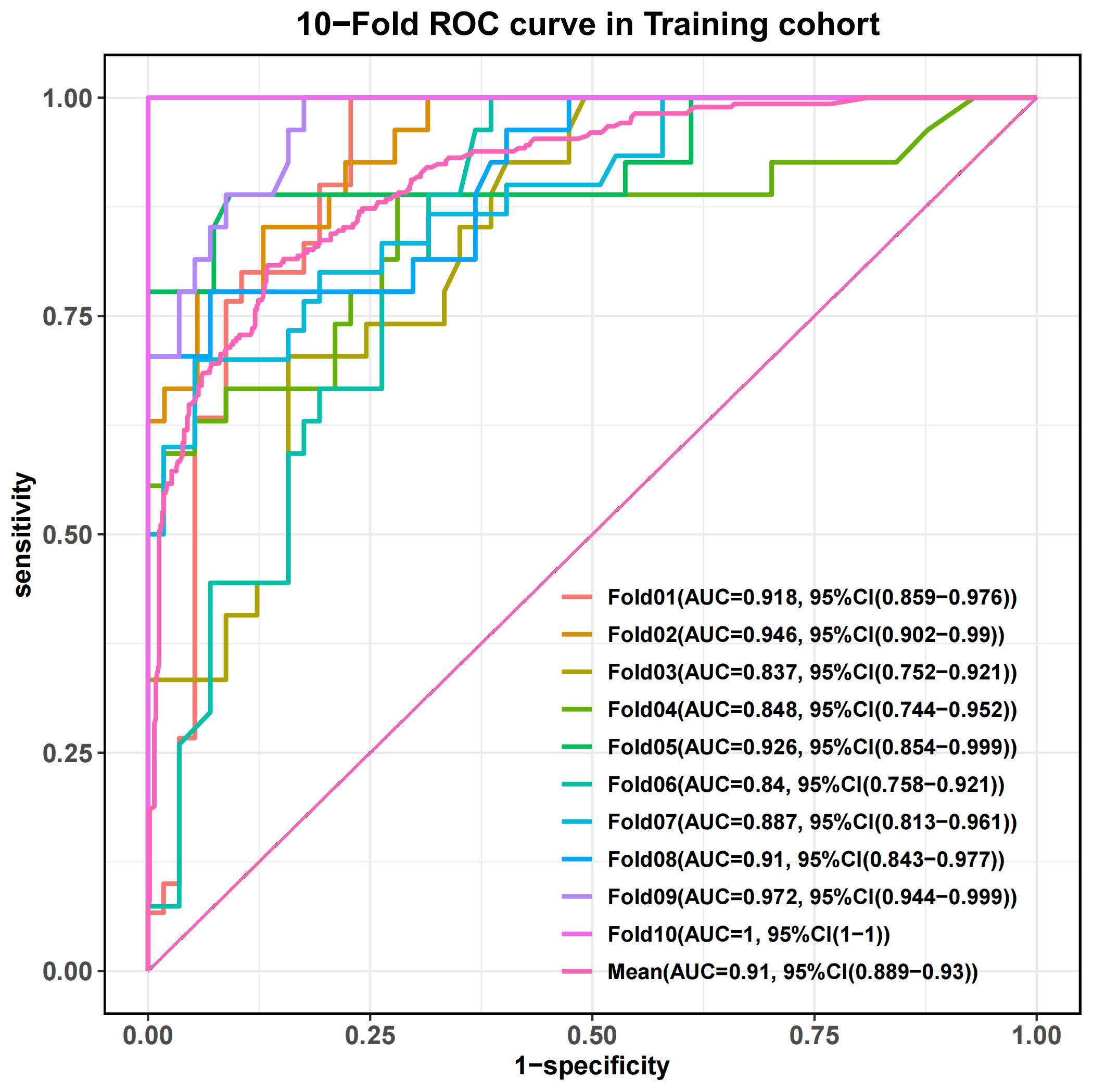


**Supplementary Materials 2** The receiver operating characteristic (ROC) curve of the random forest model undergoing ten-fold cross-validation on the training cohort demonstrates an average area under the curve (AUC) of 0.91 (95% confidence interval: 0.889-0.93). The AUC values across the folds range from 0.837 to 1.000, indicating stable and superior discriminative performance of the model on the training data
