# Supplementary material for: Development and External Validation of an Interpretable Machine Learning‐Based Prediction Model for Depressive Symptoms in Patients With Obstructive Sleep Apnea: A Multicenter Study
Source: Brain Behav. 2026 Apr 23;16(4):e71399. doi: 10.1002/brb3.71399 (PMC13103541; doi:10.1002/brb3.71399)
Supplement: Supplementary file 3 — Supplementary Materials: brb371399‐sup‐0003‐SuppMat.docx [file BRB3-16-e71399-s001.docx]

**Supplementary Materials 3 Sensitivity analyses of random forest model performance in the external validation set.**

**Model robustness to potential predictor redundancy was examined by alternately excluding one variable from each correlated pair (AHI vs. OSA severity; sleep quality vs. total sleep time) and reassessing discrimination, calibration, and net benefit. (A–D) ROC curves; (E–H) calibration plots; (I–L) decision curve analysis (DCA) for the four exclusion scenarios, ordered as: AHI excluded/OSA severity retained; OSA severity excluded/AHI retained; sleep quality excluded/total sleep time retained; total sleep time excluded/sleep quality retained.**
