# Supplementary figures and images for: Development and External Validation of an Interpretable Machine Learning‐Based Prediction Model for Depressive Symptoms in Patients With Obstructive Sleep Apnea: A Multicenter Study
Source: Brain Behav. 2026 Apr 23;16(4):e71399. doi: 10.1002/brb3.71399 (PMC13103541; doi:10.1002/brb3.71399)

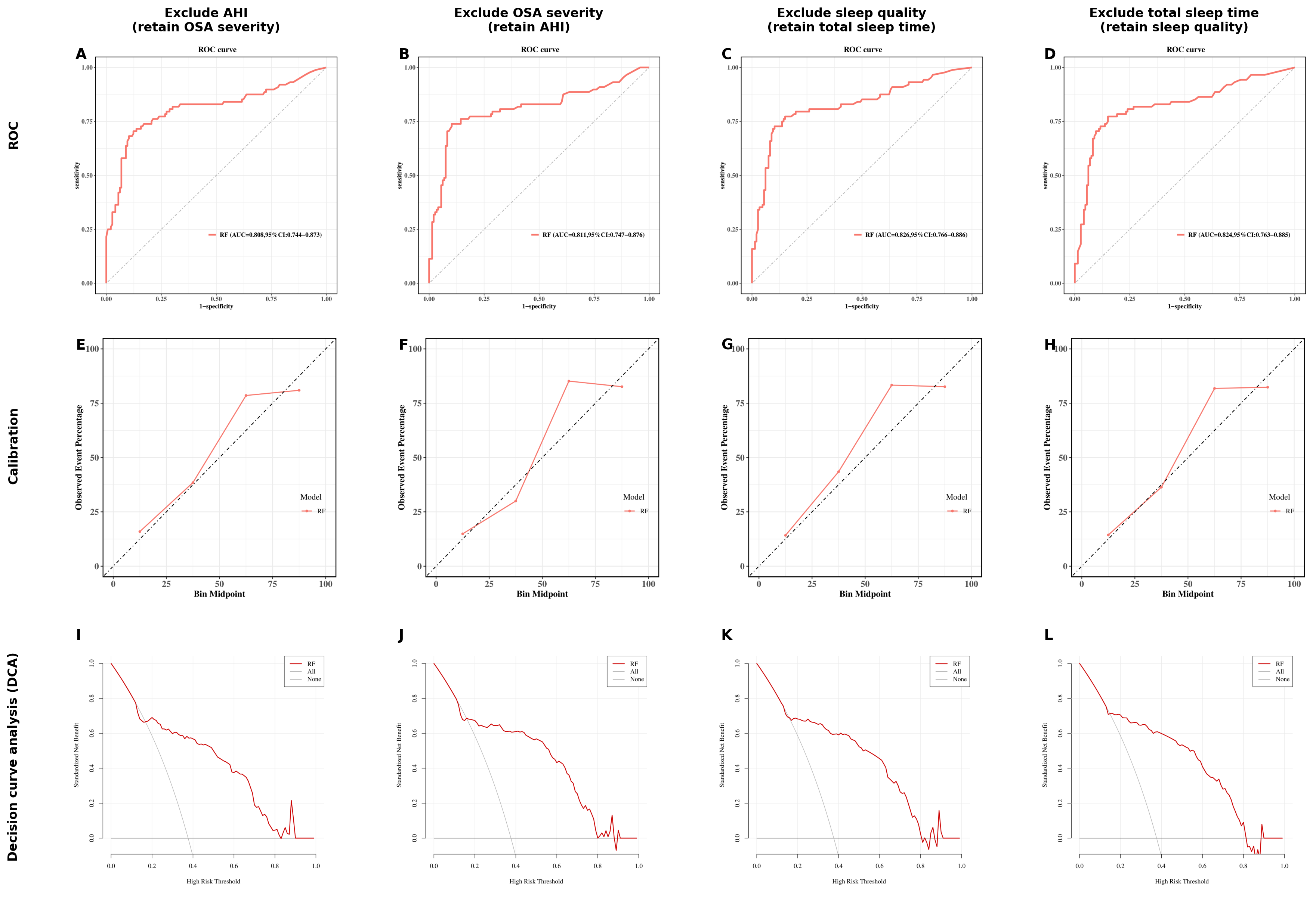

Supplement: Supplementary file 4 — Supplementary Materials: brb371399‐sup‐0004‐SuppMat.png [file BRB3-16-e71399-s002.png]
