# Supplementary material for: Development and External Validation of an Interpretable Machine Learning‐Based Prediction Model for Depressive Symptoms in Patients With Obstructive Sleep Apnea: A Multicenter Study
Source: Brain Behav. 2026 Apr 23;16(4):e71399. doi: 10.1002/brb3.71399 (PMC13103541; doi:10.1002/brb3.71399)

Supplementary Materials 4. Depressive-symptom prevalence across AHI strata (training cohort).

AHI in the training cohort ranged from 6.8 to 129.5 events/h, with a median of 27.05 events/h (IQR 21.32–43.20). The 95th percentile was 83.13 events/h, indicating that an AHI of 83 lies near the upper tail but within the observed clinical spectrum. Within the typical treatable range, depressive-symptom prevalence was 23.5% (42/179) for AHI 10–40 events/h and 28.8% (42/146) for AHI 15–40 events/h.

AHI strata (events/h)	n	Depressive symptoms(n)	Prevalence (%)	95% CI
5–<15	53	0	0.0	0.0–6.8
15–<30	130	37	28.5	21.4–36.7
30–<60	62	29	46.8	34.9–59.0
≥60	35	26	74.3	57.9–85.8

Note: P for trend across ordered AHI strata < 0.001.

Supplementary Figure S4. Prevalence of depressive symptoms across AHI strata (training cohort).

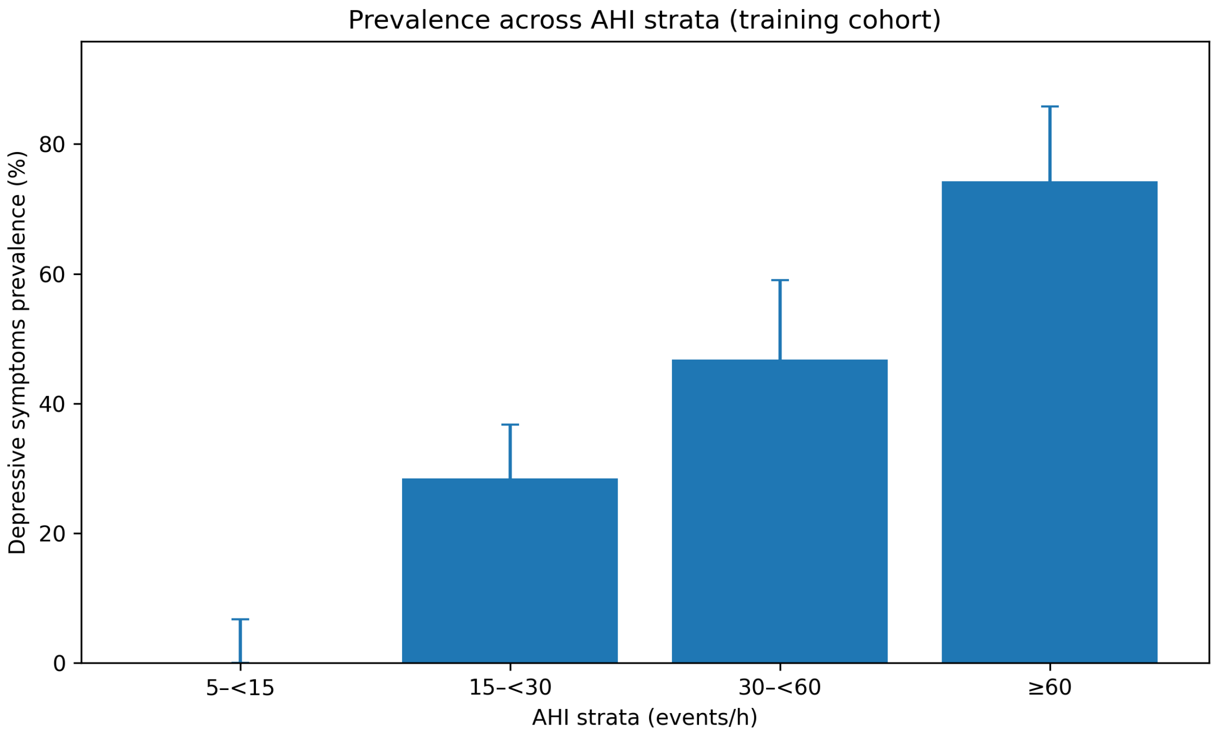

Supplement: Supplementary file 5 — Supplementary Materials: brb371399‐sup‐0005‐SuppMat.pdf [file BRB3-16-e71399-s004.pdf]
