# Supplementary material for: Development and External Validation of an Interpretable Machine Learning‐Based Prediction Model for Depressive Symptoms in Patients With Obstructive Sleep Apnea: A Multicenter Study
Source: Brain Behav. 2026 Apr 23;16(4):e71399. doi: 10.1002/brb3.71399 (PMC13103541; doi:10.1002/brb3.71399)
Supplement: Supplementary file 6 — Supplementary Materials: brb371399‐sup‐0006‐SuppMat.docx [file BRB3-16-e71399-s003.docx]

**Supplementary Materials 5 SHAP dependence plots for the predictors retained in the final random forest model**

**Supplementary Figure S1 SHAP dependence plots for the continuous predictors.**


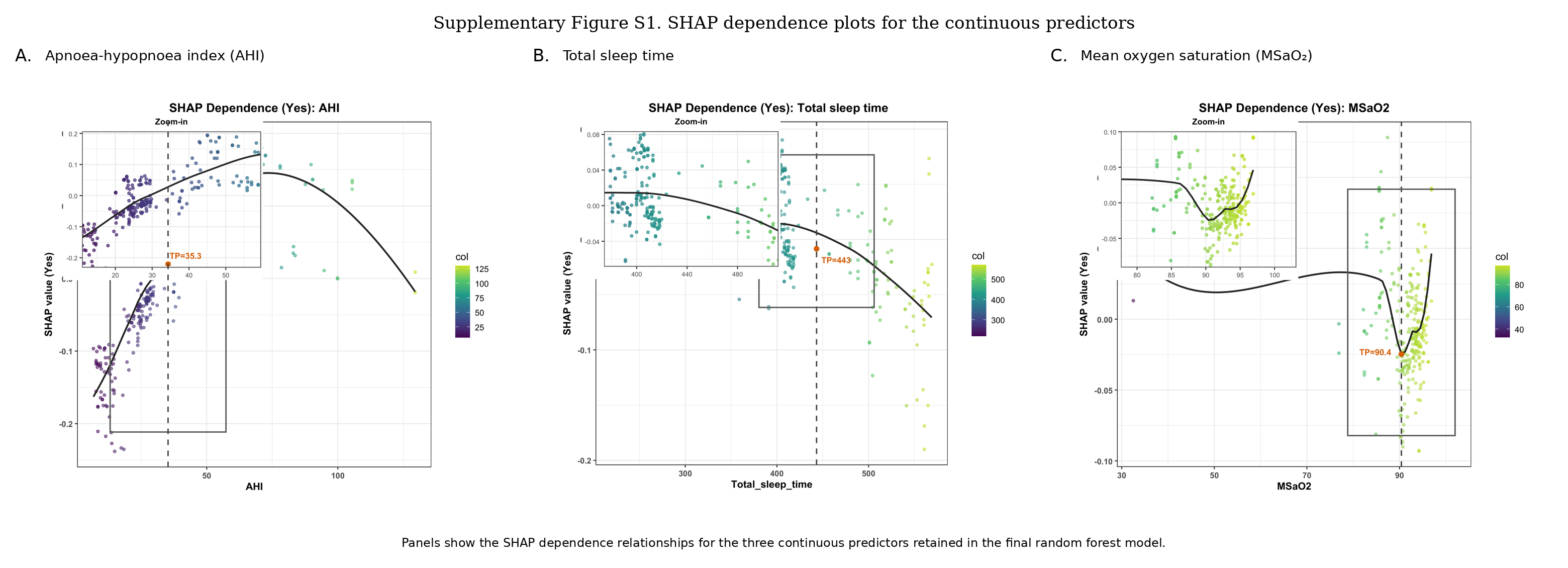


Panels A–C show the SHAP dependence relationships for apnoea–hypopnoea index (AHI), total sleep time, and mean oxygen saturation (MSaO₂), respectively.

**Supplementary Figure S2 SHAP dependence plots for the categorical predictors.**


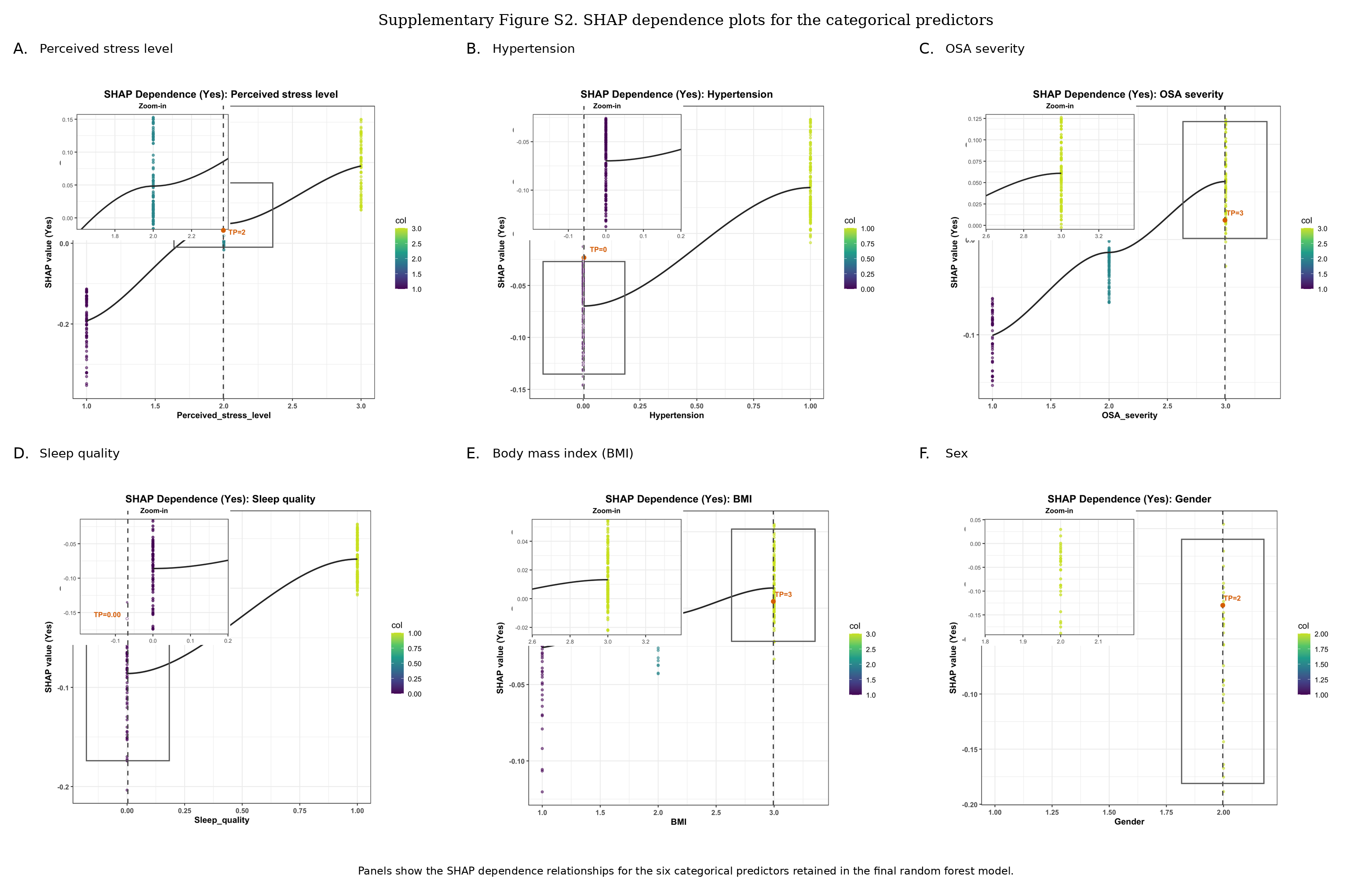


Panels A–F show the SHAP dependence relationships for perceived stress level, hypertension, OSA severity, sleep quality, body mass index, and sex, respectively.
